# Elephant (*Loxodonta africana*) Home Ranges in Sabi Sand Reserve and Kruger National Park: A Five-Year Satellite Tracking Study

**DOI:** 10.1371/journal.pone.0003902

**Published:** 2008-12-09

**Authors:** Bindi Thomas, John D. Holland, Edward O. Minot

**Affiliations:** Ecology Group, Institute of Natural Resources, Massey University, Palmerston North, New Zealand; Centre National de la Recherche Scientifique, France

## Abstract

During a five-year GPS satellite tracking study in Sabi Sand Reserve (SSR) and Kruger National Park (KNP) we monitored the daily movements of an elephant cow (*Loxodonta africana*) from September 2003 to August 2008. The study animal was confirmed to be part of a group of seven elephants therefore her position is representative of the matriarchal group. We found that the study animal did not use habitat randomly and confirmed strong seasonal fidelity to its summer and winter five-year home ranges. The cow's summer home range was in KNP in an area more than four times that of her SSR winter home range. She exhibited clear park habitation with up to three visits per year travelling via a well-defined northern or southern corridor. There was a positive correlation between the daily distance the elephant walked and minimum daily temperature and the elephant was significantly closer to rivers and artificial waterholes than would be expected if it were moving randomly in KNP and SSR. Transect lines established through the home ranges were surveyed to further understand the fine scale of the landscape and vegetation representative of the home ranges.

## Introduction

The 650 km^2^ Sabi Sand Reserve (SSR) is an association of 17 freehold game lodges and private game reserves sharing a common 50-km unfenced eastern boundary with Kruger National Park (KNP). Together, they form 20,650 km^2^ of undisturbed savanna, woodland, mountain terrain and riverine forest, and are home to 490 bird species, 147 mammals, 94 reptiles, 33 amphibians and 200 tree species [1]. The reserves are in the north east of South Africa where KNP is bordered by Mozambique to the east and Zimbabwe to the north.

At one time, the study area was a popular hunting region where elephants were heavily targeted. However, after its establishment as a South African Government Reserve in 1898, and KNP in 1923, elephants began to recolonise the area. Both KNP and SSR are managed as autonomous units with the former answerable to a conservation minister and the latter to private shareholders.

The fence between KNP and SSR was dropped in 1993 after which elephant numbers in SSR increased rapidly from 60 to 1,398 (2.15/km^2^) by 2007, an average annual increase of 13.8%. This compares with 3.9% per annum in KNP where elephant numbers during the same period rose from 7,834 to 13,050 (0.65/km^2^) [2]. The increase in elephant numbers has led some scientists to fear that continued growth will result in tree canopy destruction that may exacerbate reductions in species richness of birds and other taxa [3], [4].

In 1989, Whyte [3] concluded that effective elephant management policies in KNP should be supported by a better understanding of elephant movement patterns. Consequently, Whyte used radio transmitters to study the movements of 29 adult KNP elephants during a seven-year period to 1996 [3]. He tracked each elephant for an average period of four years and, using an average of 10 location points each year, identified home ranges varying from 45 km^2^ to 1800 km^2^ and observed that movements were not always confined within individual parks. In 2006, the paucity of elephant movement data were highlighted by a panel of scientists who reported that a more precise understanding of elephant movements are required if successful management programmes are to be developed [4].

In our study we tracked the daily movements of the study animal for five years to further understand the location, size and inter-annual variability of home ranges; identify travel corridors between parks; and consider how the resources within the reserves influence movement.

## Materials and Methods

**Figure 1:**
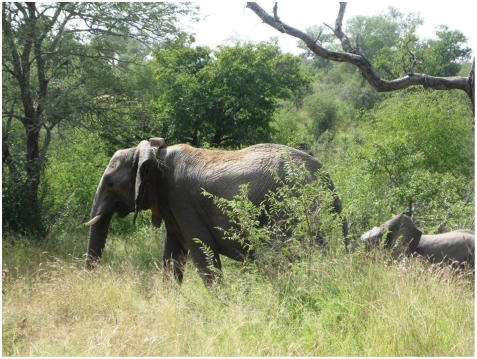
Study elephant with satellite tracking collar (Photo J Holland).

In conjunction with the Sabi Sand ecologist and staff from KNP's Scientific Services and Veterinary Wildlife Services, a breeding herd cow was identified and darted from a helicopter on the 26 September 2003 and a satellite collar attached. Before the batteries of the tracking collar expired, the animal was retagged on 15 August 2006. Observations of daily movements since the retagging showed no obvious signs of stress as there were no changes in the daily movement patterns. In previous studies, the matriarchs of the family group were selected [3], however, because these are the oldest animals and susceptible to a higher mortality [3], we selected a younger, lactating cow, estimated to be 24 years old with a small calf at foot (see Figure 1). The study animal is a member of a matriarchal group of three adult and four juvenile elephants.

We tagged the elephant on a cool day to avoid overheating and death of the animal. The tranquiliser dart was fired from a modified shotgun at the rump using a 24 cm×7 ml aluminium syringe dart with a 3 mm ‘collared’ needle. The drug combination used to tranquilize the lactating cow was short-acting Azaperone (Janssen Pharmaceutica) and the analgesic etorphine hydrochloride or M99 (Norvartis) with Diprenorphine or M-5050 as an anaesthetic and antidote respectively [3], [5]. Upon the elephant becoming recumbent, the rest of the matriarchal group was herded off to a safe distance by the helicopter.

The helicopter team was accompanied by a ground crew to roll the immobilized elephant on its side in the event it collapsed on its haunches after tranquilising. The pressure from the weight of the elephant upon the diaphragm and sternum may have injured or killed the animal [5]. The elephant's exposed eye was covered with its ear to protect it from direct sunlight and dust and the trunk was extended to ensure the animal breathed comfortably.

### Equipment

A combination of satellite receivers and a GPS transmitter were used to monitor the elephant's movements. The Inmarsat 3 F1 is a third generation satellite (1996) covering the whole of Africa, Australia and Middle East [6].

The tracking unit attached to the elephant had a GPS receiver and a VHF radio transmitter incorporated into the collar. The unit on the elephant was set to obtain and transmit a single location signal at noon (local time) each day. We monitored the period 26 September 2003 to 30 July 2008, equating to approximately 1,750 tracking-days.

The location data were mapped and analysed using ArcGIS® ArcMap® 9.2 (Environmental Systems Research Institute, Redlands, California, USA), with Spatial Analyst® and Tracking Analyst® extensions. Home range area was determined by calculating the Minimum Convex Polygon (MCP) using Animal Movement Analyst Extension (AMAE) [7]. A MCP is known to inflate the actual area occupied by the animal because it includes outliers. According to Kenward [8], however, a MCP including all locations is the most widely used home range estimator allowing for meaningful comparisons between home ranges of different studies. This being the case, we calculated home range using a MCP with all the locations for our study animal and, to allow for a more conservative estimate, recalculated it with 95 and 50% of the locations. Outliers were removed with AMAE utilising the harmonic mean method [9].

Weather data were obtained from the South African Weather Bureau station at Skukuza. The station is located within the study area and recorded average annual rainfall and temperature of 541 mm and 23.8°C respectively during the five-year period. Summer is the rainy season and winter is the dry period when the animals become increasingly dependant upon waterholes and manmade dams. This is true for most African national parks [10]–[12].

### Habitat study

**Figure 2:**
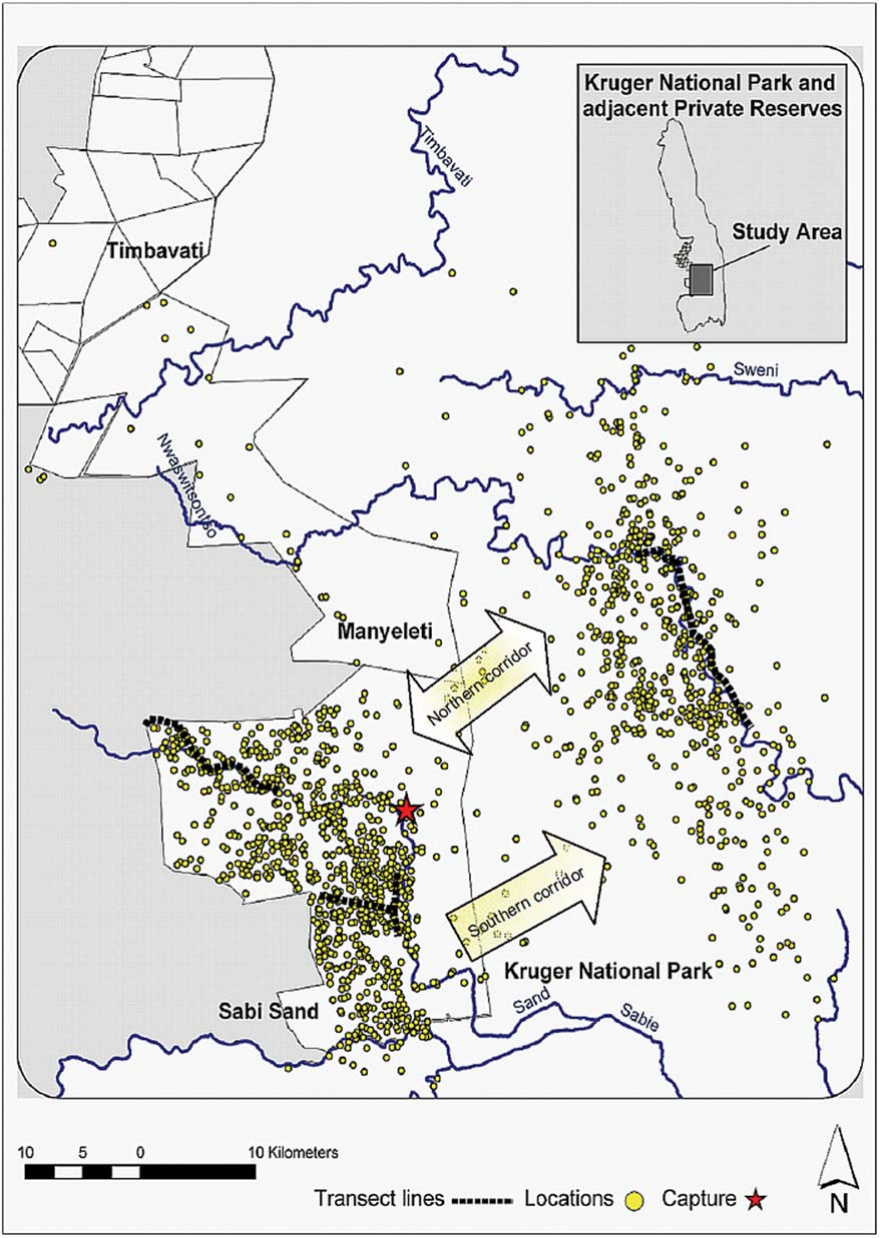
Study area and daily locations of study elephant. Map of KNP, South Africa showing the locations of daily GPS fixes from the study animal obtained from September 2003 to July 2008.

We mapped daily location points to identify core winter and summer home ranges through which transect lines were surveyed to further understand the fine scale of the predominant landscape and vegetation representative of the home ranges. The SSR and KNP transect lines (Figure 2) were 28 km and 20 km long respectively and sampling was conducted at one kilometre intervals. Vegetation and landscape attributes within a 30-meter radius of each study site were recorded. The SSR home range area was shown to be more biologically diverse. Dominant tree species in both areas include knob thorn *Acacia nigrescens*, sickle bush *Dichrostachys cinerea* and russet bushwillow *Combretum apiculatum*. Guinea grass *Panicum maximum* is ubiquitous to both home range areas.

## Results and Discussion

### Habitat use

The elephant's daily location points are mapped in Figure 2, showing the concentrations of location points within each park.

We found that the elephant in this study did not use the available habitat randomly, instead developing a strong preference for a specific habitat while others were seldom, if ever, used. These findings are similar to those of Ntumi, van Aarde, Fairall, and de Boer [13].

**Figure 3:**
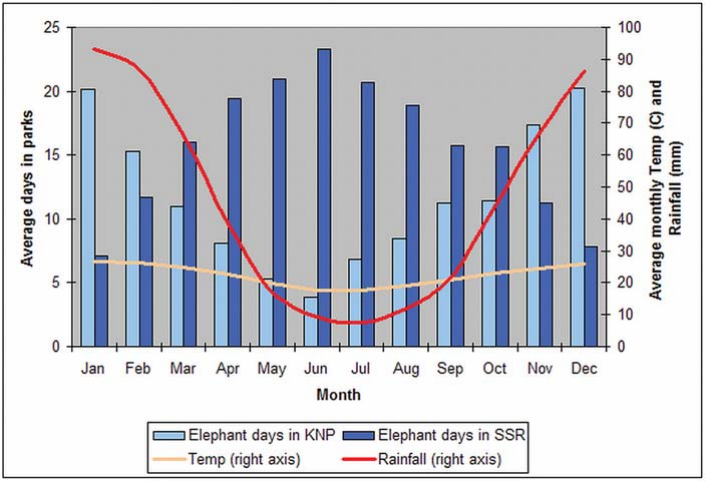
Average monthly occupancy rates. The average monthly occupancy of the study elephant within Kruger National and Sabi Sand Parks compared to average monthly temperature (°C) and rainfall (mm) from September 2003 to July 2008.

Seventy two percent of all positions recorded during the summer months (December, January and February) were located within KNP and 77% of winter positions (June, July and August) were located within SSR. Average monthly visitation rates to KNP over the five-year period peaked at 20 days during December and January before the herd moved to the well-watered SSR in June when visitation rates are highest (23 days) and coincide with lowest average rainfall and temperature (Figure 3).

**Figure 4:**
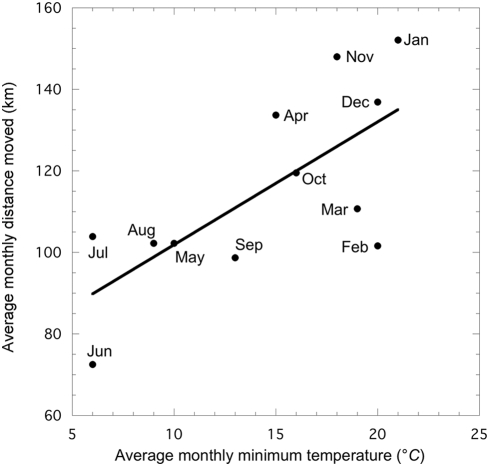
Average monthly distance and minimum temperature. Distance is based on a single GPS location taken at noon each day and average minimum temperature for the period 1960–1990 from the Skukuza weather station.

Using the distance between consecutive mid-day locations as a proxy for daily distance travelled by the elephant, we found that it walked an average of 127 km per month during summer compared with 101 km per month during winter (paired-sample *t* = 2.25, df = 3, *P*<0.05). Whilst it is difficult to attribute changes in behaviour to specific variables, or combinations of variables, we found that the study elephant's movement increased as temperature increased (r = 0.71; *P*<0.001; Figure 4). This is most likely because the coldest months are also the driest and, given that elephants need to drink every day or two [4] they move to their winter home range where there is a high density of waterholes, so less movement is necessary.

Within SSR, the study elephant barely utilised the eastern boundary with KNP and a small pocket in the south west of SSR (Figure 2). This may be attributable to the limited number of eastern border waterholes [14] and the large human presence near the unutilised south west pocket of the SSR.

In June 2007, landscape and vegetation transects were conducted in the core home ranges and 33 tree and 21 grass species were identified in the SSR and KNP home ranges respectively. The SSR home range was more biologically diverse with 30% of the trees and 57% of the grasses found in SSR not represented in the KNP transect samples (Table 1).

**Table 1:** Dominant tree and grass species identified in vegetation transect through elephant home ranges in Kruger National and Sabi Sand Parks (June 2007) 1 ^,^ 2.

Dominant species	Soils and topography	SSR *(n = 28)*		KNP *(n = 20)*	
		No.^3^	%	No.	%
**TREES**					
*Euclea divinorum* Magic guarri	Mostly found in the brackish flats in granite & alluvial soils along river courses. Generally growing in pockets among other tree species, in thorn scrub, hillsides & woodland.	19	68	5	25
*Acacia nigrescens* Knob thorn	Usually occurs in groups. The largest trees are found in the flood-plains & shrub form is common in the gabbro & basalt areas.	16	57	1	5
*Dichrostachys cinerea* Sickle bush	Prefers clay-like soils but also found on all soils & close to rivers & brackish flats. Also along roads due to increased run-off.	16	57	11	55
*Combretum hereroense* Russet bushwillow	Most often seen around pans, rocky areas & sometimes on stream banks. Usually occurs in closely associated groups.	14	50	13	65
*Ziziphus mucronata* Buffalo thorn	Found everywhere but prefers brackish flat & koppie, open woodland, often in alluvial soils & on termite mounds.	12	43	0	0
*Sclerocarya birrea* Marula	Common throughout the Lowveld, growing on all soil types.	12	43	7	35
*Combretum apiculatum* Red bushwillow	Often found on granite crests. As with the mopane, the red bushwillow is one of the most abundant trees in area.	8	29	1	5
*Lonchocarpus capassa* Apple-leaf	Common in most parts, grows on all soil types, tallest & most plentiful on alluvial plains & on river & stream banks.	7	25	13	65
*Acacia nilotica* Scented thorn	Prefers brackish soils near rivers & drainage lines. Also found on clay soils.	7	25	4	20
*Terminalia sericea* Silver cluster-leaf	Found in granite area, prefers deep, well-drained, sandy soils. Prolific on mid-slope seep-lines where it grows in dense groups. Common in higher rainfall areas.	6	21	0	0
*Grewia monticola* Silver raisin bush	Small to medium size deciduous tree 2–10 m. Occurs over wide range of altitudes in riverine fringes & open woodland - often on termite mounds.	5	18	5	25
*Spirostachys africana* Tamboti	Occurs on all soil types, common in the Lowveld. Often in groups of a few big trees along rivers or streams in the brackish flats.	5	18	5	25
*Peltophorum africanum* African weeping wattle	Grows best in lower altitudes in wooded grassland & on well-drained sandy soils, but occurs on all soil types in area.	5	18	4	20
*Diospyros mespiliformis* Jackal berry	Grows along most river courses & bigger streams at lower altitude woodlands. Often found growing away from drainage lines & on termite mounds.	5	18	4	20
**GRASSES**					
*Panicum maximum* Guinea grass	Tufted perennial, grows on all soils; damp places along fertile soil; shade of trees & along rivers.	17	61	16	80
Heteroptogon contortus Spear grass	Fast-growing grass that likes well-drained stony soils; open areas; twisted seed-heads are often seen along roadsides.	9	32	11	55
*Digitaria eriantha* Finger grass	Tufted perennial that grows in open areas & on moist soils - especially in sandy areas.	8	29	6	30
*Pogonarthria squarrosa* Sickle grass	Perennial that grows in well-drained sandy soils. Common in disturbed places - an indicator of poor, sandy soils, old lands.	8	29	0	0
*Perotis patens* Cat's tail grass	Tufted perennial that grows on disturbed soils, often in poor sandy soils and dry exposed sites.	5	18	7	35
*Setaria Sphacelata Torta* Creeping bristle grass	Creeping perennial grass that likes granitic, well-drained soils. Good soil conservation grass that forms runners that bind soil.	5	18	11	55
*Dactyloctenium australe* L.M. grass	Creeping perennial that thrives in shade in sandy soil. Popular lawn grass in Lowveld.	4	14	0	0
*Themeda triandra* Red grass	Tufted perennial that grows on basalt, gabbro and dolerite and undisturbed grassland areas.	4	14	0	0
*Chloris virgata* Feather top chloris	Variable annual grows in shade but prefers open country. Not drought tolerant.	3	11	0	0

1 Fyvie [21].

2 A total of 33 tree and 21 grass species were identified in SSR and KNP.

3 Refers to the number of times species were identified in the 28 SSR transect location sites.

This may explain the elephant's preference for the undulating granite/gneiss and gabbro plains of SSR during winter while the summer, rain-charged rivers of the Karoo Sediment home range plains in KNP may be one of the reasons that the elephant targets this landscape and vegetation type. During the rainy season the elephant selects from a narrower choice of habitats. At this time of the year, the plants in KNP elephants' diet decrease [13]. Codron et al. [15] have shown that elephants in the study area tend to become dependant upon grass during summer, with tree-felling and debarking of larger trees starting in winter when the grass dries and the elephants begin eating woody plants. This response is intensified during draught periods [4], [16]. We agree with Ntumi et al. [13] who stress that most elephants favour closed canopy habitat types like riparian thickets and vegetation types associated with water.

**Figure 5:**
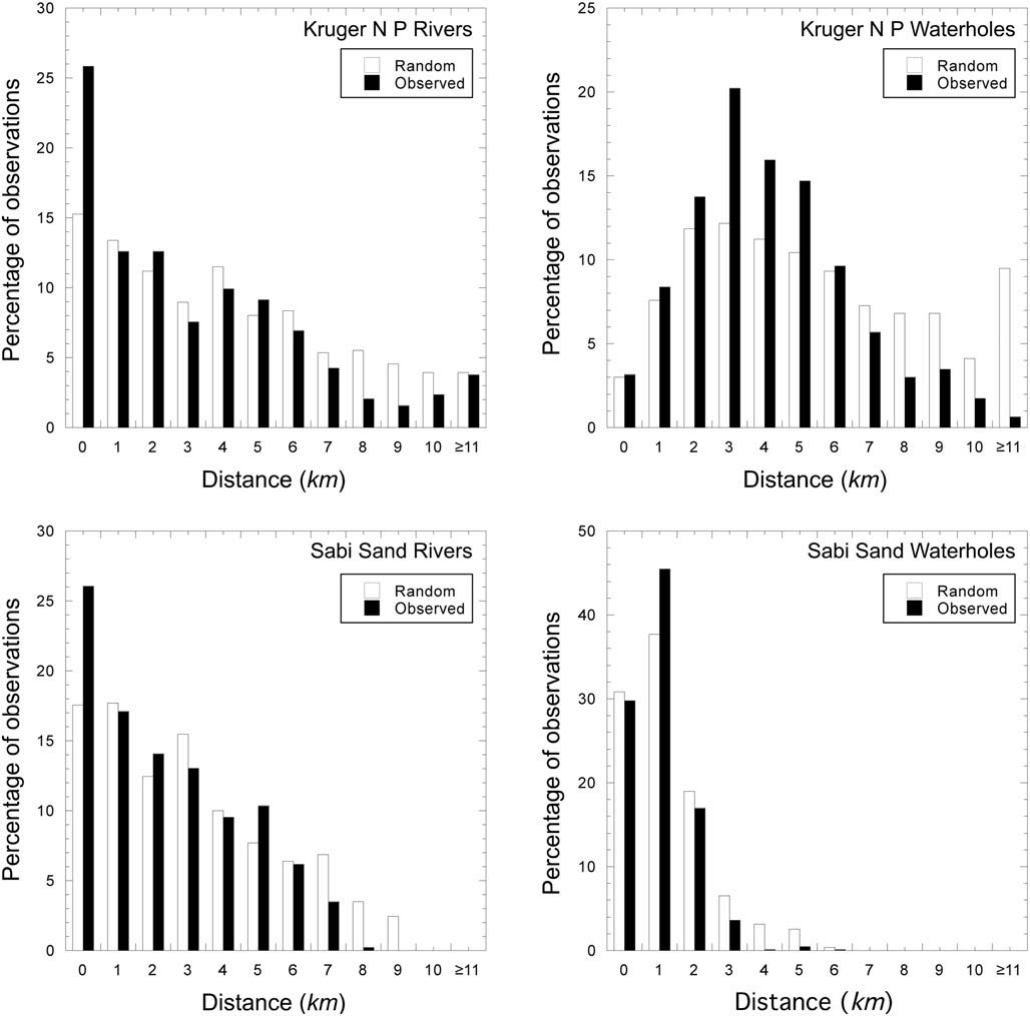
Observed and random location distances from water sources. Random points were generated within the 95% MCP for the KNP and SSR home ranges. For both observed elephant locations and random points, the distance to the nearest water, either waterhole or river, was calculated.

Our findings concur with those of Smit, Grant and Whyte [17], namely, that the herd occurred closer to water sources more frequently than would be expected if they were randomly distributed. The observed locations were significantly closer to waterholes and rivers in both KNP and SSR than random locations (Figure 5).

By 2007, SSR had 376 waterholes or 0.58 waterholes per km^2^ and 2.15 elephants per km^2^ compared with a KNP density of 0.65 elephants per km^2^. This, in conjunction with improved winter browsing and a well-distributed, reliable water supply make the SSR an attractive winter destination. Elephants in KNP consume varying proportions of browse to grass in different seasons [15] and it may be that the wider diversity of woody plants in the study animal's winter home range allows elephants to utilise this resource more efficiently after grass production drops off following the dry summer. During the wet summer, elephants increase their grass consumption to around 50% and then change over to the less varied summer diet [15], [18].

### Home range

The distance between the core summer and winter home ranges was 32 km. The 95 and 50% MCP combined home ranges for KNP and SSR were 2,244 km^2^ and 783 km^2^ respectively. Comparable results from KNP from Whyte's [3] study of 29 radio-tracked elephants give 90% MCPs ranging from 45 to 1,800 km^2^. While our results align with his upper estimates his overall results will tend to be low because he used only 10 locations per year.

The elephant's five-year SSR winter home range was 308 km^2^ and its annual average home range during the same period was 131 km^2^. This shows that, whilst the elephants move back to the same broad geographic area each year, only 40% is utilised during any one year. The five-year KNP summer home range was 1,139 km^2^ and its annual average home range for the same period is 424 km^2^. The annual average home range within SSR is in line with the findings of Fairall [19] for the same reserve (<200 km^2^). Also our estimates for the KNP home range is similar to reports by Whyte (523 km^2^) and Hall-Martin (436 km^2^) in 2001 and 1984 respectively [13].

In Table 2 the details of the study animal's movements between SSR and KNP over the five-year period are presented, revealing that only eight of the 26 habitation periods were less than a month in duration. The cow moved between the two home ranges up to three times a year. The average annual winter home range size in SSR is 195 km^2^ compared with 331 km^2^ for its summer counterpart in KNP and, as can be expected, the home range increases with the time the elephant spends in each reserve (r = .78, *p*<0.002 for SSR; r = .61, *p*<.03 for KNP). From tracking the elephant's movement between the two reserves, a northern and southern corridor were identified (Figure 2). Between November 2003 and April 2008, the corridors were traversed on 25 occasions with the busier northern corridor used for 78% of the crossings. Notably, all the movements from KNP to SSR were through the northern corridor.

**Table 2:** Park and corridor usage of SSR and KNP.

Sabi Sand Reserve				Kruger National Park			
N^1^	Departure Date^2^	95% MCP (km^2^)^3^	Departure corridor^4^	N	Date	95%MCP (km^2^)	Departure corridor^4^
37^5^	3 Nov 03	115	Southern	7	10 Nov 03	127	Northern
45	25 Dec 03	220	Northern	108	11 Apr 04	485	Northern
189	16 Oct 04	322	Southern	91	13 Jan 05	514	Northern
196	29 Jul 05	447	Northern	31	29 Aug 05	177	Northern
7	5 Sep 05	20	Northern	59	3 Nov 05	230	Northern
27	30 Nov 05	180	Southern	37	6 Jan 06	341	Midway
6	12 Jan 06	68	Northern	54	8 Mar 06	547	Northern
128	12 Jul 06	288	Northern	50	1 Sep 06	235	Northern
84	24 Nov 06	340	Southern	83	15 Feb 07	188	Northern^6^
46	1 Apr 07	149	Northern	22	24 Apr 07	242	Midway
160	1 Oct 07	137	Northern	19	20 Oct 07	192	Northern
14	3 Nov 07	35	Northern	71	11 Jan 08	624	Northern^7^
98	18 Apr 08	209	Northern	47	5 Jun 08	403	
**Ave MCP**		195				331	
**5-year MCP**		308				1139	

1 Number of days within the park before movement.

2 Date that elephant started journey to other home range.

3 The MCP (km^2^) is based on locations obtained inside the relevant park calculated after arrival date from previous home range.

4 Refer to Figure 2 for corridor location.

5 From 26 September onwards.

6 Entered corridor via Manyeleti.

7 Entered corridor via Timbavati.

### Management implications

This study followed a single female, and consequently her group of three adults and four juveniles, for a period of five years. While this long duration is not a substitute for extensive replication, this is the first KNP/SSR elephant to be tagged with a satellite collar and, being the longest continuous study of its kind in the area, the results provide the first insight into within- and between-season movements. It is known that female elephants live and travel within distinct matriarchal groups each led by closely related matriarchs who may be sisters or cousins and, together, the groups form part of a wider composition known as a ‘bond group’. Therefore, movement of one adult female could be extrapolated to the movements of a matriarchal group. Two similar elephant tracking studies are currently being undertaken by Thomas, Minot and Holland in the same study area and the data from the first 18 months of this on-going research support the findings of this study. Namely, that both move between the two parks, summer MCP home ranges are larger than winter home ranges and both are utilised in the same manner as reported in this study.

This long time series has enabled us to report on the reciprocal importance of KNP and SSR to the elephant and its attendant herd and that since the fence between the two reserves was dropped, the elephants consistently rely upon KNP for summer grazing and SSR for winter grazing and water. It has also enabled us to identify possible important northern and southern corridors between the reserves. This, combined with the rising number of elephants in both reserves signals the importance of ongoing co-operation between wildlife managers from both reserves.

In 1999, SANParks approved a new policy for managing the KNP elephant population based upon the park being divided into zones and managed according to biodiversity impacts rather than on fixed elephant numbers [2], [3]. These were designed to broadly conform to home ranges that were identified using radio-collared elephants of herds in selected zones [2], [20]. However, the radio data were limited to less than one location point per month and, notwithstanding the valuable contribution of this early research to understanding elephant movements at a broad level, it would not have been possible to identify specific movement corridors between home ranges; isolate shorter visits made by animals to home ranges; identify movement patterns between home ranges; and map the full extent of an elephant's home range.

Future management plans could be more comprehensive by recognizing that the two areas must be managed as a single unit. From the results of our study, we conclude that the boundary recommended for the southern high-impact region [3] would only accommodate the elephant's summer home range. The proposed ‘high-intensity’ elephant zone does not include the elephant's SSR winter home range area. Both KNP and SSR share similar challenges associated with overpopulation, the provision of artificial waterholes, and monitoring and evaluation of flora and fauna. Therefore, a co-operative management plan taking into account seasonal elephant use of both parks, and the corridors between them, should be a priority.

This study illustrates the advantages of long-term continuous monitoring of wildlife in both better understanding their seasonal ecology and formulating management plans based on their habitat requirements throughout the year.
